# Multiscale mechanisms of nutritionally induced property variation in spider silks

**DOI:** 10.1371/journal.pone.0192005

**Published:** 2018-02-01

**Authors:** Sean J. Blamires, Madeleine Nobbs, Penny J. Martens, I-Min Tso, Wei-Tsung Chuang, Chung-Kai Chang, Hwo-Shuenn Sheu

**Affiliations:** 1 Evolution & Ecology Research Centre, School of Biological, Earth & Environmental Sciences D26, The University of New South Wales, Sydney, Australia; 2 Graduate School of Biomedical Engineering, Samuels Building F25, The University of New South Wales, Sydney, Australia; 3 Department of Life Science, Tunghai University, Taichung, Taiwan; 4 National Synchrotron Radiation Research Centre, Hsinchu, Taiwan; Scientific Research Centre of the Slovenian Academy of Sciences and Art, SLOVENIA

## Abstract

Variability in spider major ampullate (MA) silk properties at different scales has proven difficult to determine and remains an obstacle to the development of synthetic fibers mimicking MA silk performance. A multitude of techniques may be used to measure multiscale aspects of silk properties. Here we fed five species of Araneoid spider solutions that either contained protein or were protein deprived and performed silk tensile tests, small and wide-angle X-ray scattering (SAXS/WAXS), amino acid composition analyses, and silk gene expression analyses, to resolve persistent questions about how nutrient deprivation induces variations in MA silk mechanical properties across scales. Our analyses found that the properties of each spider’s silk varied differently in response to variations in their protein intake. We found changes in the crystalline and non-crystalline nanostructures to play specific roles in inducing the property variations we found. Across treatment *MaSp* expression patterns differed in each of the five species. We found that in most species *MaSp* expression and amino acid composition variations did not conform with our predictions based on a traditional *MaSp* expression model. In general, changes to the silk’s alanine and proline compositions influenced the alignment of the proteins within the silk’s amorphous region, which influenced silk extensibility and toughness. Variations in structural alignment in the crystalline and non-crystalline regions influenced ultimate strength independent of genetic expression. Our study provides the deepest insights thus far into the mechanisms of how MA silk properties vary from gene expression to nanostructure formations to fiber mechanics. Such knowledge is imperative for promoting the production of synthetic silk fibers.

## Introduction

Spider major ampullate (MA) silk is nature’s toughest materials [1]. Accordingly, there is considerable interest in the creation of materials that mimic its performance [2]. Nevertheless, attempts to recombine, amplify and spin spider silk proteins have not produced fibers with properties resembling those of naturally spun silk [3,4]. One reason for the inability to produce such fibers is that the properties of MA silk are highly variable and the mechanisms inducing this variation have never been delineated from nano to macro scales [5].

Researchers can determine the consequences of gene expression on the functional properties of structural proteins by either switching genes on and off and observing the consequences in the secreted proteins, or by observing the function of the proteins produced in different organisms with varying levels of expression of a particular gene [5]. Indeed, using such approaches has informed us how and why certain genes cause specific ailments in humans and other animals [5,6]. Progress has been recently made into our understanding of spider silk genes and their expression patterns [7–11]. Likewise, significant insights have been gained on silk production, spinning, and its engineering [11–15]. However, because no single study has holistically examined the consequences of gene expression on protein structure and silk functional properties, it is not known why spiders spin silks with such exceptional properties and, more importantly, why silk properties vary so much between and within individual spiders [11,14]. Experimentally switching silk related genes on and off within individual spiders is not yet achievable, so observing the function of silk proteins in different spiders with varying levels of expression of particular genes appears the best way forward.

MA silk properties have traditionally been thought to be the product of the combined expression of genes coding for two proteins (called spidroins); major ampullate spidroin 1, or MaSp1, and major ampullate spidroin 2, or MaSp2 [11,16] (with the genes called *MaSp1* and *MaSp2*). The secondary structures of the spidroins are considered critical for silk performance [17,18]. MaSp1 consists of repeating polyalanine, (GA)_*n*,_ (GGX)_*n*_ and (A)_*n*_ amino acid motifs (G = glycine, A = alanine and X = other amino acids). These motifs form cassettes that combine to promote the formation of crystalline β–sheet nanostructures in the assembled fibers [18,19]. The MaSp2 protein on the other hand has been thought to consist of multiple (GPGXX)_*n*_ motifs (where P = proline), and predicted to form disordered type II β-turns and similar non-crystalline nanostructures [20]. Collectively, the various nanostructures are thought to combine and provide MA silk with its great strength and extensibility [21]. Since MaSp2 has long been predicted to contain (GPGXX)_*n*_ sequences, the proline composition of MA silk was considered a reliable indicator of *MaSp2* gene expression [22,23]. The ratio of *MaSp1*: *MaSp2* expression is variable among and between spider species, presumably because the MaSp2 protein is metabolically costly to synthesize so may be differentially expressed. Variation in the ratio of the two spidroins has been traditionally thought to bring about variations in nanostructure formations leading to variations in the mechanics of the spun fibres [11].

The recent mapping of the *Nephila clavipes*’ spidroin genome [10] has caused researchers to rethink much assumed knowledge. We now understand that, at least in *N*. *clavipes*: (i) individual spiders express multiple versions of the *MaSp* genes at different loci, potentially leading to several MaSp1 and MaSp2 proteins appearing in the spun silks, (ii) the different spidroin cassettes and motifs are shared and partitioned among the different proteins, and (iii) other spidroins, e.g. AcSps, appear in the major ampullate gland, so can provide additional cassettes and motifs that may form components of MA silk [10,16,24]. For these reasons, MA silk amino acid compositions may not necessarily reflect the *MaSp1*: *MaSp2* expression, possibly explaining why nanostructure formations can vary within individual spider silks independent of *MaSp1*: *MaSp2* expression [25–27].

Any study aspiring to understand how spidroin gene expression influences MA silk nanostructure formation must reliably measure the size, density, orientation, and distances between the crystalline and non-crystalline nanostructures in the silk proteins in addition to genetic expression. Techniques such as small angle X-ray scattering (SAXS), wide angle X-ray scattering (WAXS), nuclear magnetic resonance (NMR), Fourier transform infrared (FTIR), Raman and circular dichroism (CD) spectroscopy, and transmission electron microscopy, have been used to examine the nanostructures of MA silk’s proteins [28–31]. Of these only synchrotron-derived SAXS and WAXS can explicitly and reliably measure nanoscale variability in silk crystallinity, and the size, density, orientation, and distance between individual crystalline and non-crystalline nanostructures [32,33]. For instance, SAXS derived parameters such as the meridional peak and long period can be used to elucidate nanostructure alignment in the silk’s amorphous and lamellar regions [34–36]. WAXS on the other hand can be used to determine silk crystallinity, and the size, density, and orientation, of crystalline nanostructures by examining the scattering pattern and diffraction angles (*θ*) at specific high intensity diffraction peaks [28,37,38]. For spider silk the diffraction peaks at the (200), (120) or (002) regions identified on two dimensional WAXS images are of particular interest as they are associated with scattering from crystalline β–sheets [37,39–41]. SAXS and WAXS accordingly are tools appropriate for measuring and classifying silks based on the size, alignment, and distances, between and within crystalline and non-crystalline nanostructures [37,41,42].

X-ray scattering analyses of the MA silks of different spider species has found that the micro-arrangement of silks from spiders in the Araneoidea clade are relatively conserved across the group [41]. The mechanical properties of Araneoid spider MA silks, nonetheless, may vary considerably between species and individuals across environments and loading preconditions [43]. For instance, silk spun by spiders walking along a surface is not as stiff as that spun by free falling spiders [43,44]. Much of this variability is considered a consequence of changes in the friction acting on the silk as it exits the valve during spinning since this induces the amorphous region proteins to further self-align [30,43]. The properties of the crystalline nanostructures can nonetheless also vary within individual spider silks across loading conditions. For example, spiders exposed to winds of different strength produce silks differing in nanocrystal density, which affects the ultimate strength of the fibers [25]. Exposing spiders to strong wind induces silk extensibility and ultimate strength to change in the same direction [25,45,46]. Simulations and experiments have shown that variations in glandular pH, salts and shear stress during spinning induce the poly-alanine residues to undergo α-helix→ β-sheet phase transitions, which enhances silk strength [47–50]. These types of phase transitions could explain the enhancement of ultimate strength from spiders exposed to high wind. Moreover, it has been shown that the additional enhancement of extensibility in the silks of spiders exposed to strong wind could be a consequence of spinning under a static load, since this induces the amorphous region proteins to move more freely relative to each other [46].

An additional experiment found that the spider *Nephila pilipes* produces stronger and tougher MA silk when fed a high protein diet than when deprived of protein [27]. Subsequent WAXS analyses found that, like wind exposed spiders [25], changes in silk crystal density explains some of the enhanced strength [27]. Nevertheless, unlike wind exposed spiders, a combination of changes in crystallinity and nanostructural orientation in the amorphous region were also prevalent [27]. In both instances, the silk nanostructures seem to vary independent of *MaSp1*: *MaSp2* expression, although only indirect measures (i.e. amino acid composition) of *MaSp* expression was made [25,27]. Different mechanisms at different scales seem to be responsible for nutritionally induced spider silk property changes compared to wind induced property changes. However, no study has holistically examined the consequences of gene expression on silk proteins and protein structure and, ultimately, silk functional properties to understand the mechanisms inducing nutritionally induced property variation in spider silks at multiple scales.

Here we performed an examination of nutritionally induced MA silk property variation by running experiments similar to those described by Blamires et al. [27]. However, we used five species of Araneoid spiders: *Argiope keyserlingi*, *Eriophora transmarina*, *Latrodectus hasselti*, *Nephila plumipes* and *Phonognatha graeffei*, and directly measured spidroin expression using quantitative real-time PCR (RT-PCR) techniques. Specifically, our experiments aimed to answer two persistent and problematic questions about spider silk property variability: (1) Do the silk nanostructures and mechanical properties of different spiders respond similarly to variations in spidroin expression? And (2) what are the relative contributions of changes to amino acid compositions and nanostructures in inducing spider silk mechanical property variation? To answer the first question, we performed silk tensile tests, SAXS/WAXS analyses, amino acid determinations, and gene expression analysis, for the abovementioned spiders and compared the results across species and treatments. To answer the second question, we pooled the mechanical property, nanostructural, and amino acid compositional data across species and constructed predictive models.

## Materials and methods

### Ethics statement

Ethical clearance was not required to perform this research. Capture permits were not required under New South Wales law as all collections were made outside of protected areas. We confirm that the collection locations were not privately owned, and we did not collect any endangered or protected species.

#### General methods

**Fig 1:**
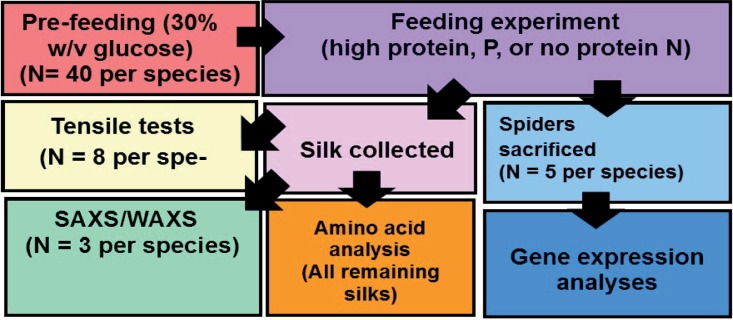
Flow diagram of the methods implemented to examine the consequences of protein deprivation on MA silk gene expression, protein nanostructures, and mechanical properties for five species of spiders.

Fig 1 overviews the methods implemented to, collectively, examine the consequences of gene expression on silk protein nanostructures and functional properties.

#### Spider collection and pre-feeding

We collected, per species, 40 adult female *A*. *keyserlingi*, *E*. *transmarina*, *L*. *hasselti*, *N*. *plumipes* and *P*. *graeffei* from locations between Sydney and Ballina, New South Wales, Australia, during trips made between October 2014 and January 2015. To ensure spiders of approximately equal size within species were used and that no gravid spiders were collected, we measured each spider’s body length and width to ±0.1 mm using digital Vernier calipers (Caliper Technologies Corp., Mountain View, CA, USA) and mass to ±0.001g using an electronic balance (Ohaus Corp., Pine Brook, NY, USA) upon collection, and discarded any particularly large or heavy (>50% above the mean) individuals. We returned all the required spiders to the laboratory at the University of New South Wales, Sydney, where they were placed in 115 mm (wide) x 45 mm (high) plastic circular containers. The containers had perforated wire mesh lids with a 20 mm long slit cut into them using a Stanley knife to facilitate feeding with a 50 μl micropipette. We pre-fed the spiders 20 μl (*A*. *keyserlingi*, *L*. *hasselti* and *P*. *graeffei*) or 50 μl (*E*. *transmarina* and *N*. *plumipes*) of a 30% (w/v) glucose solution daily over five days (for details see [27,51]) to standardize the diet of all spiders prior to experimentation. We reweighed the spiders after the pre-feeding treatment and any individuals who lost > 50% of their initial mass (one *A*. *keyserlingi*, one *L*. *hasselti* and three *N*. *plumipes*) were discarded.

### Feeding experiments

We randomly divided the remaining 40 or so spiders per species equally into two groups and fed them either one of two solutions over 10 days: a protein solution (P) or protein deprived solution (N). The protein solution used to experimentally increase protein intake was identical to that used by Blamires et al. [27], i.e. a mixture of 10g of a 10% albumin solution with 6g of sucrose in 60ml of water. The protein deprived solution was 8g of sucrose in 30 ml of water. We fed the spiders by placing a measured droplet of solution onto their chelicerae using a 20 μl micropipette (see [27,51]). As protein and carbohydrates contain approximately similar energy densities (~4kJ g^-1^), solutions of similar energy concentrations were fed to all spiders. After completing the feeding experiment we re-weighed all of the spiders and any that lost > 20% of their mass during the experiment (i.e. one *A*. *keyserlingi*, two *L*. *hasselti* and *P*. *graeffei*, and five *N*. *plumipes*) were not used for any of the subsequent experiments.

### Silk collection

After completing the feeding experiment and subsequent re-weighing, we anaesthetized each spider (N = 185 spiders; 38 *A*. *keyserlingi*, 40 *E*. *transmarina*, 37 *L*. *hasselti*, 32 *N*. *plumipes*, and 38 *P*. *graeffei*) using CO_2_ and carefully pulled a single MA silk fiber from their spinnerets using tweezers. We collected a thread of silk from eight spiders per treatment per species for the determination of mechanical properties as follows.

We connected a revolving headframe to an electronic spool. We then attached a 240 mm long x 40 mm wide cardboard strip with six 10 mm x 10 mm square holes punched at 30 mm intervals to the headframe. Double sided sticky tape was stuck onto the cardboard at the border of the holes. A silk thread was pulled over the headframe and stuck to the sticky tape. The headframe was rotated once at 1m min^-1^ while ensuring the silk traversed all of the square holes and adhered to the pieces of tape. The strip was then removed from the headframe and a drop of water based glue applied at the position where the silk attached to the tape. Another frame of equal size with identically positioned holes punched into it was placed on top. The two strips were squeezed together with forceps ensuring that they were tightly stuck together. We then cut the strip in the regions between the holes perpendicular to the silk thread, thus leaving six 10mm x 10mm frames each holding parts of a single thread of silk. The procedure was repeated for every individual used from each of the five species. Accordingly, 48 frames were collected per treatment per species, i.e. 6 frames x 8 individual threads (see [26,52] for details).

We collected silk from a further three individuals per treatment per species for SAXS/WAXS analyses. We did this by spooling their silk onto 3 mm x 1 mm titanium frames containing 0.5 mm x 0.5 mm windows (see [52]) for ~1 h (*A*. *keyserlingi*, *L*. *hasselti* and *P*. *graeffei*) or ~2 h (*E*. *transmarina* and *N*. *plumipes*). We assumed that the amount of silk extracted was approximately the entire store of silk from the spider’s major ampullate glands. We collected between 1000–2000 rounds of silk across the windows of each frame. We have previously found this amount of silk to be adequate for attaining quality scattering from 5-35keV synchrotron X-ray sources [25,27,52].

We collected silk from the remaining 75 spiders [16 *A*. *keyerslingi* (8 each from the N and P treatment), 18 *E*. *transmarina* (9 N and 9 P), 15 *L*. *hasselti* (7 N and 8 P), 10 *N*. *plumipes* (4 N and 6 P) and 16 *P*. *graeffei* (8 N and 8 P)] to determine their amino acid compositions. We wrapped the silk threads around a glass tube connected to the electronic spool spun at 1m min^-1^for ~1–2 h. This approximated the collection of the store of silk from the spider’s glands, so any variations in amino acid composition within individual threads were accounted for. All silks were extracted under controlled temperature (~25°C) and humidity (~50% R.H.) in still air, so reeling speed and post-spin environment did not influence the subsequent chemical or mechanical property measurements.

### Mechanical property determination

One randomly selected frame of silk from each thread (i.e. one of the six frames of silk collected per spider) was used to ascertain the width of the thread [26,52] so we could calculate the cross-sectional area of the individual threads used in the ensuing tensile tests. We taped the frames to a microscope slide and examined and photographed them under air immersion at 1000x magnification using a polarized light microscope (CKX41, Olympus, Tokyo, Japan) connected to a SPOT Idea 5 Mp digital camera (Spot Imaging Solutions, Sterling Heights, MI, USA). The images were digitized using the program Spot Basic 4.7 (Spot Imaging Solutions, Sterling Heights, MI, USA) and the width of each thread determined as a mean of 12 measurements using the program Image J (NIH, Bethesda MD, USA).

We performed the following tensile tests under controlled temperature (~25°C) and humidity (~50% R.H.) in still air within 10 days of silk collection. We placed each of the cardboard frame-mounted silks for each species within the grips of an Instron 5543 tensile testing machine (Instron Machines, Melbourne, Australia) with a ~2μN resolution [26]. We ensured that the grips held the silks firmly at the upper and lower frame edges. The left and right sides of the frames were cut away and the silks stretched at a rate of 0.1 mm s^-1^ until the fiber ruptured.

True stress (σ) and strain (ε) were derived from the following equations:

$$\sigma =\frac{F}{A}$$

and

$$\varepsilon =\log_{e}\frac{L}{L_{0}}$$

where F is the force applied to the specimen, A is the cross-sectional area of the thread calculated from the thread diameter assuming a constant thread volume, L is the instantaneous length of the fiber at a given extension value and L_0_ is the original gage length of the fiber. Stress *vs* strain curves were determined for each silk tested by a standard trapezoidal method from which we calculated the following mechanical properties using both Bluehill 3.0 (Instron Machines, Melbourne, Australia) and Microsoft Excel 2010: (1) ultimate strength; or the stress at rupture, (2) extensibility; or the strain at rupture, (3) toughness; the area under the stress strain curve, and (4) Young’s modulus (stiffness); the slope of the stress-strain curve during its initial elastic phase.

### SAXS/WAXS experiments

Small-angle X-ray scattering (SAXS) procedures were performed at the end station of beamline BL23A SWAXS of the Taiwan Light Source, National Synchrotron Radiation Research Center (NSRRC), Hsinchu, Taiwan. Pre-tests were performed using polyethylene and silver behenate to calibrate the scattering intensity and wave vector sensitivity, respectively. The samples were placed 3659 mm from the incident 15 keV X-ray beam (λ = 0.8265 Å) and were exposed to the beam for 10–60 s depending on the measured signal intensity. The scattered radiation was captured using a Pilatus 1M-F area detector and two dimensional SAXS images generated. From these images the scattering intensity was obtained and intensity *vs* scattering vector (*q*) plots generated using the program Albula (Dectris, Baden-Dättwil, Switzerland). Where *q* was ascertained by the equation:

$$q=4\pi \lambda^{-1}\sin\theta$$

where λ is the wavelength of the incident X-ray beam (λ = 1.033Å) and *θ* is the scattering angle [53].

We then calculated the meridional peak following Balta-Calleja and Vonk [54], from which we estimated the long period (*L*) using the equation [54–56]:

$$L=2\pi /q_{m}$$

where the *q*_m_ is the integrated position of the meridional peak ascertained by scanning the intensity *vs q* plots along the equatorial direction [34].

We performed WAXS procedures immediately upon completion of the SAXS procedures using the same 30 silk samples at the end station of beamline BL01C2 at NSRRC, Hsinchu, Taiwan. We used the same samples because we were interested in measuring a combination of nanostructural properties within the crystalline, amorphous and lamella regions of the same silk threads. We thus first performed a series of pre-tests to establish that the short exposure time (10–60 s) of the SAXS procedures was unlikely to damage the silk nanostructures and affect the WAXS measurements.

We placed the samples 300 mm from the 12keV incident beam and exposed them to X-rays for 10–60 min depending on the pre-measured signal intensity. Beam size was confined by a collimator 0.5 mm in diameter. Scattered radiation was detected by a Mar 345 imaging plate and two dimensional diffraction images generated for each silk sample using the program Fit2D (ESRF, Grenoble, France). From the diffraction images we calculated the diffraction angle (*θ*), intensity peaks (*I*_x_), and 2*θ* full width and half width maximum intensities (FWHM) of the (200) and (120) diffraction peaks and the so called amorphous halo.

We subsequently calculated:

(i) The relative crystalline intensity ratios (*I*_200_/ *I*_120_) with *I*_200_ and *I*_120_ representing the sum of the intensity peaks at the (200) and (120) peaks respectively [57].
(ii) The crystallinity index, *X*_c_, calculated according to Grubb and Jelinski [58], and
(iii) Herman’s orientation function, f_c_, using the equation [42]:

$$f_{c}=(3\left\{\cos^{2}\theta \right\}‑1)/2$$

where φ is the angle between the c axis and the fiber axis, {cos^2^φ} is the azimuthal width at the (200) and (120) diffraction peaks determined using the equation [42]:

{cos2φ}=1−A{cos2φ1}‑B{cos2φ2}

where A = 0.8 and B = 1.2.

### Amino acid composition determination

We weighed all of the silk samples designated for amino acid composition analysis to the nearest 0.001 mg on an electronic balance (Pioneer PA214C, Ohaus, Pine Brook NJ, USA), before submergence in 99% hexoflouro-isopropanol solvent (500 μ*l* of per mg of silk) within 1 ml Eppendorf tubes. The samples were then hydrolyzed in 6 mol *l*^-1^ HCl for 24 h at 115°C. Molar percentage compositions of glutamine, serine, proline, glycine, and alanine, the amino acids representing ~90% of the total amino acids in the MA silks of most spiders [59], determined using an Alliance Systems (Waters, Rydalmere NSW, Australia) high performance liquid chromatography column [60] at the Australian Proteomic Analysis Facility, Sydney.

### Spidroin gene expression analysis

At the end of silk collecting five randomly selected spiders per treatment and species were sacrificed by lethal exposure to CO_2_and their major ampullate glands dissected as described by Jeffrey et al. [61]. The glands and a sample of the remaining abdomen were immediately lysed with RNase free mini pestles in QIAzol Lysis Reagent and mRNA extracted using an RNeasy Plus Universal RNA extraction kit (Qiagen, Düsseldorf, Germany). In order to prevent any DNA contamination we used a gDNA Eliminator Solution provided with the extraction kit to remove all genomic DNA. The extracted mRNA was eluted to 30–35 μl and we measured the concentration extracted using a NanoDrop 1000 Spectrophotometer (Thermo Fisher Scientific, Wilmington, DE, USA). A mean concentration of 1376.4 ± 239.04 ng/μl RNA was extracted from all samples and did not differ substantially between species. Mean absorbance ratios of the samples were 2.24 at 260/280 nm, and ranged between 1.86 and 2.38, and 1.97 at 260/230 nm and ranged between 1.22 and 2.27. Absorbance ratios in this range are considered acceptable as ‘pure’ for single stranded RNA [62].

All of the mRNA samples were diluted to 1000ng/μl. We took 12.5 μl subsamples of the diluted mRNA for reverse transcription to cDNA using an Advantage reverse transcription kit for PCR (Clontech, Clayton Vic, Australia). The reverse transcription and PCR activation procedures were carried using Eppindorf Mastercycler (Eppindorf, Mamburg, Germany) qPCR machines, following the recipe outlined by the reverse transcription kit handbook [63]. A “buffers only” (i.e. no RNA and no Reverse Transcriptase) solution was included in the analyses as a negative control. We included the spider’s abdomens in the gene expression analysis for normalization against background expression of *MaSp* transcripts in other silk glands or abdominal tissue [10]. All procedures were replicated three times for each individual spider.

We used the *Drosophila rufa* Glycerol-3-phosphate dehydrogenase (g3pdh) gene as a “housekeeping” reference gene for our RT-PCR analyses, as is common practice in gene expression analyses [64,65]. However, since the g3pdh gene primers were designed from *Drosophila* spp., there may have been unintended amplification biases [66] across species.

We diluted all of the cDNA eluent to 200 ng/μl, checking (using a NanoDrop 1000 Spectrophotometer) that the 260:280nm absorbance ratio were all between 1.5 and 1.8 before sending 10 μl samples to the Ramaciotti Centre for Genomics, University of New South Wales, for Fluidigm quantitative RT-PCR gene expression analyses [67]. Fluidigm RT-PCR gene expression analysis utilizes dual-labelled probes designed to hybridize a complementary region of the cDNA for real-time amplification. These probes contain a fluorescent reporter dye on the 5' base, and a quencher on the 3' base, whose intensity increases proportional to the number of probe molecules cleaved [67].

We used published C-terminal domain sequences for *MaSp*1 and *MaSp*2 from *Argiope trifasciata* (hereon called *MaSp1*a and *MaSp2*a) and *Latrodectus hesperus* (hereon called *MaSp1*b and *MaSp2*b) [7,68–71] to order primers for the RT-PCR analyses (see Supporting Information, S1 Table for sequences and accession numbers) using the Fluidigm online assay designer [67]. We converted the threshold cycle (C_T_) values (see S2 Table) derived by the RT-PCR analyses to 2^-ΔΔC^_T_ values, which were averaged for each individual spider across the three technical replicates, following Schmittgen and Livak [62].

While recent genomic work has shown that some spider species may possess more than two *MaSp* loci, with some paralogs exhibiting different expression patterns across different silk glands [10,68,72], further verification of multiple *MaSp* loci across the Araneoid clade awaits. Since our objective here was to identify whether shifts in amino acid compositions can be attributable to shifts in *MaSp1*: *MaSp2* expression and not verify or refute the multiple loci hypothesis, we used the abovementioned procedures to ascertain the across treatments expression patterns of just two *MaSp1* and *MaSp2* paralogs.

### Analyses

For each species we used separate single-factor (two treatment levels: protein deprived and protein fed) multivariate analyses of variance (MANOVAs) and Fisher’s Least Significant Difference post-hoc analyses to determine whether the mean (± 1 standard error): (1) mechanical properties (ultimate strength, extensibility, toughness, and Young’s modulus), (2) nanostructures (*L*, 2*θ* FWHM of the (200) and (120) diffraction peaks and amorphous halo, *I*_200_*/ I*_120,_ X_c_, and f_c_), (3) mole compositions of glutamine, serine, proline, glycine, and alanine, and (4) spidroin (*MaSp1a*, *MaSp1b*, *MaSp2a*, and *MaSp2b*) expression. We used additional univariate (treatment) ANOVAs to individually compare the *MaSp1a*, *MaSp1b*, *MaSp2a*, and *MaSp2b* 2^-ΔΔC^_T_ values between treatments for each species. We log_10_ or arcsine (amino acid composition data) transformed any data that failed Levene’s heterogeneity tests.

To ascertain the influences of nanostructures and amino acid compositional variations on silk mechanical properties we pooled the data for all species and constructed multiple regression models. We used the mechanical properties that our MANOVAs found to differ across treatments as the response variables and any nanostructural parameters or amino acid compositions that our MANOVAs found to differ across treatments as the predictor variables. Species and treatments were assigned as continuous predictor variables. A large number of predictor variables, interactions, and intercept terms were likely so we considered linear regression models to be too complex for interpretation. We therefore derived Y = β_0_ + β_1_(*x*_1_) + β_2_(*x*_2_)… β_*n*_(*x*_*n*_) + ε_*i*_ additive regression models for each response variable, where β_0_ is the population intercept, β_1_, β_2_. β_*n*_ are the regression coefficients associated with the predictor variables *x*_1_, *x*_2_… *x*_*n*_ and ε_*i*_ is the random error term associated with the *i*th observation [73]. We checked all data for normality, linearity, homoscedasticity and singularity using Q-Q and scatterplots prior to constructing the models.

## Results

An overall summary of the results for the five species of spider is provided in Table 1.

**Table 1:** Summary of the consequences of protein deprivation on silk properties for the five species examined.

	*Argiope keyserlingi*	*Eriophora transmarina*	*Latrocedtus hasselti*	*Nephila* *plumipes*	*Phonognatha graefei*
Mechanical properties	↑thread width ↑extnsibility	↓extensibility	—	↑ultimate Strength ↑toughness	↑ultimate Strength ↑toughness
Crystalline structures	↓crystallinity	—	—	—	—
Non-crystalline structures	↑lamellar alignment	—	—	↓lamellar alignment ↑amorphous alignment	—
Amino acid composition	↓Gly, ↓Ala	—	—	↑Pro	↑Pro
*MaSp* expression	Upregulate *MaSp1*a	Downregulate *MaSp1*a Upregulate *MaSp1*b	Upregulate *MaSp1*a, *MaSp2*a, *MaSp2*b Downregulate *MaSp1*b	Downregulate *MaSp1*a, *MaSp2*a	Downregulate *MaSp1*a Upregulate *MaSp2*a

### Does the silk nanostructures and mechanical properties of different spiders respond similarly to variations in spidroin expression?

We found that protein feeding and deprivation affected silk mechanics differently among the five species examined (see Supporting Information, S3 Table). *Argiope keyserlingi*’s silk was more extensible when protein deprived than when protein fed. On the other hand *Eriophora transmarina*’s silk was less extensible when protein fed (Supplementary Material, S3A and S3B Table). Both *Nephila plumipes*’ and *Phonognatha graeffei*’s MA silks were stronger and tougher when they were protein deprived (Supporting Information, S3D and S3E Table). Wherein we found neither protein feeding nor deprivation to effect the mechanical properties of *Latrodectus hasselti*’s silk (Supporting Information, S3C Table). Comparisons of the silk thread widths across treatments found a significant difference for *A*. *keyserlingi* only (means ± SE: protein deprived spiders = 3.39 ± 0.18 μm, protein fed spiders = 2.17 ± 0.15 μm, Supporting Information, S3A Table), thus thread width differences across treatments were not responsible for any of the variations silk mechanical properties found across the five species.

**Fig 2:**
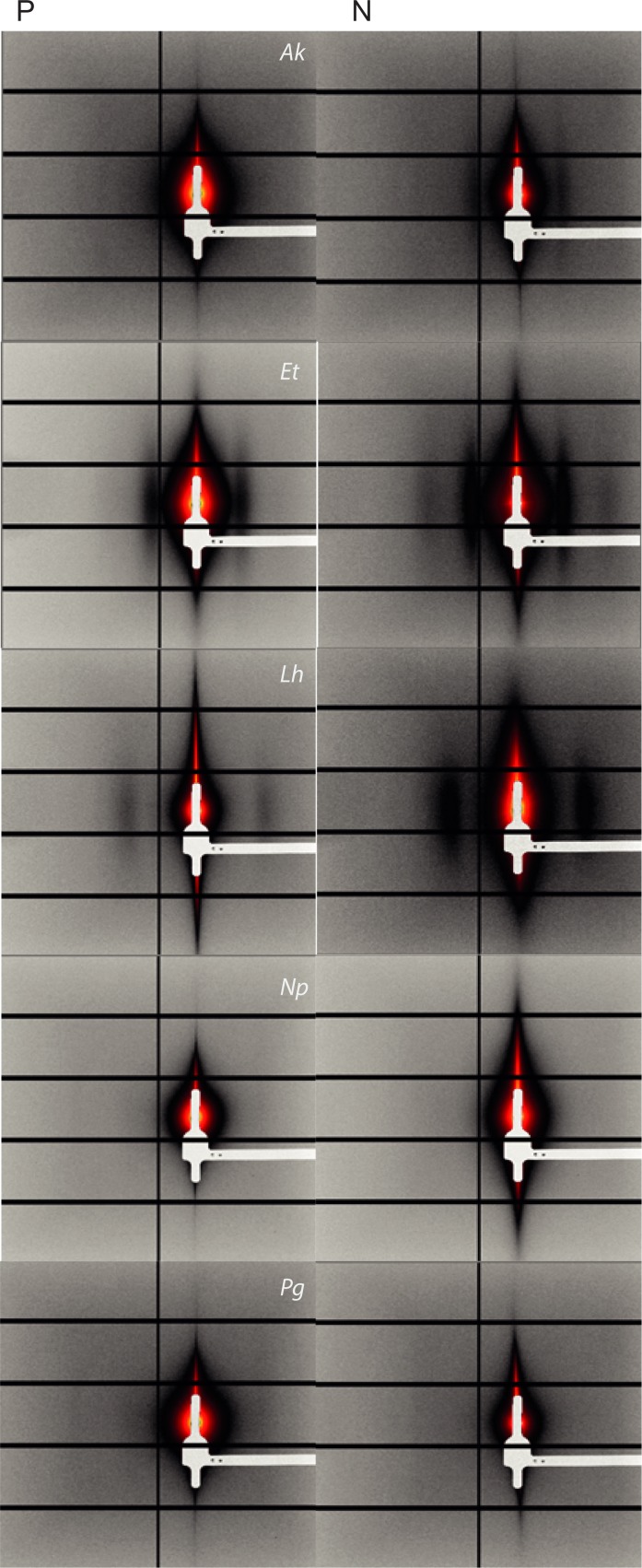
SAXS images derived for MA silks of each species of spider. Where P = protein fed and N = protein deprived treatment, *Ak* = Argiope *keyserlingi*, *Et* = *Eriophora transmarina*, *Lh* = *Latrodectus hasselti Np* = *Nephila plumipes*, *Pg* = *Phonognatha graeffei*.

**Fig 3:**
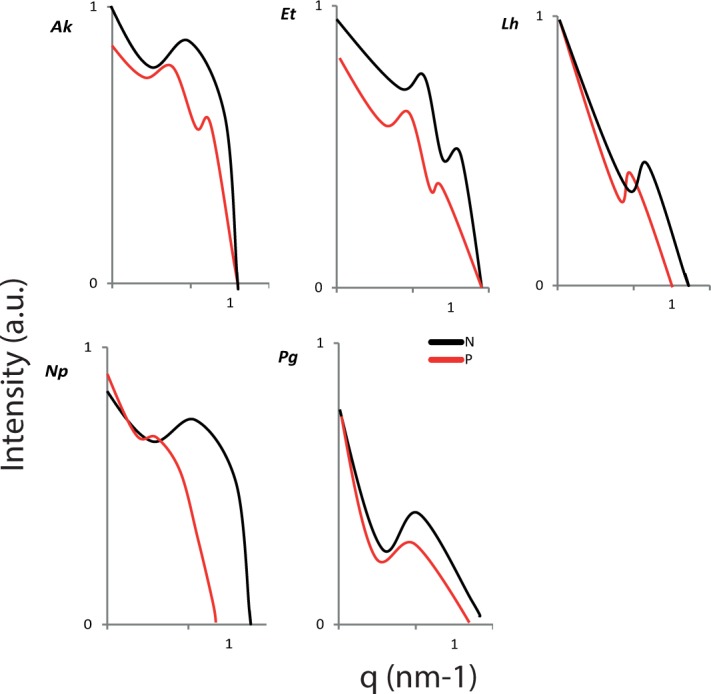
SAXS derived intensity *vs* waveband parameter (*q*) plots for the MA silks each of the 5 species’ MA silk. Where P = protein fed and N = protein deprived treatments, *Ak* = Argiope *keyserlingi*, *Et* = *Eriophora transmarina*, *Lh* = *Latrodectus hasselti Np* = *Nephila plumipes*, *Pg* = *Phonognatha graeffei*.

**Fig 4:**
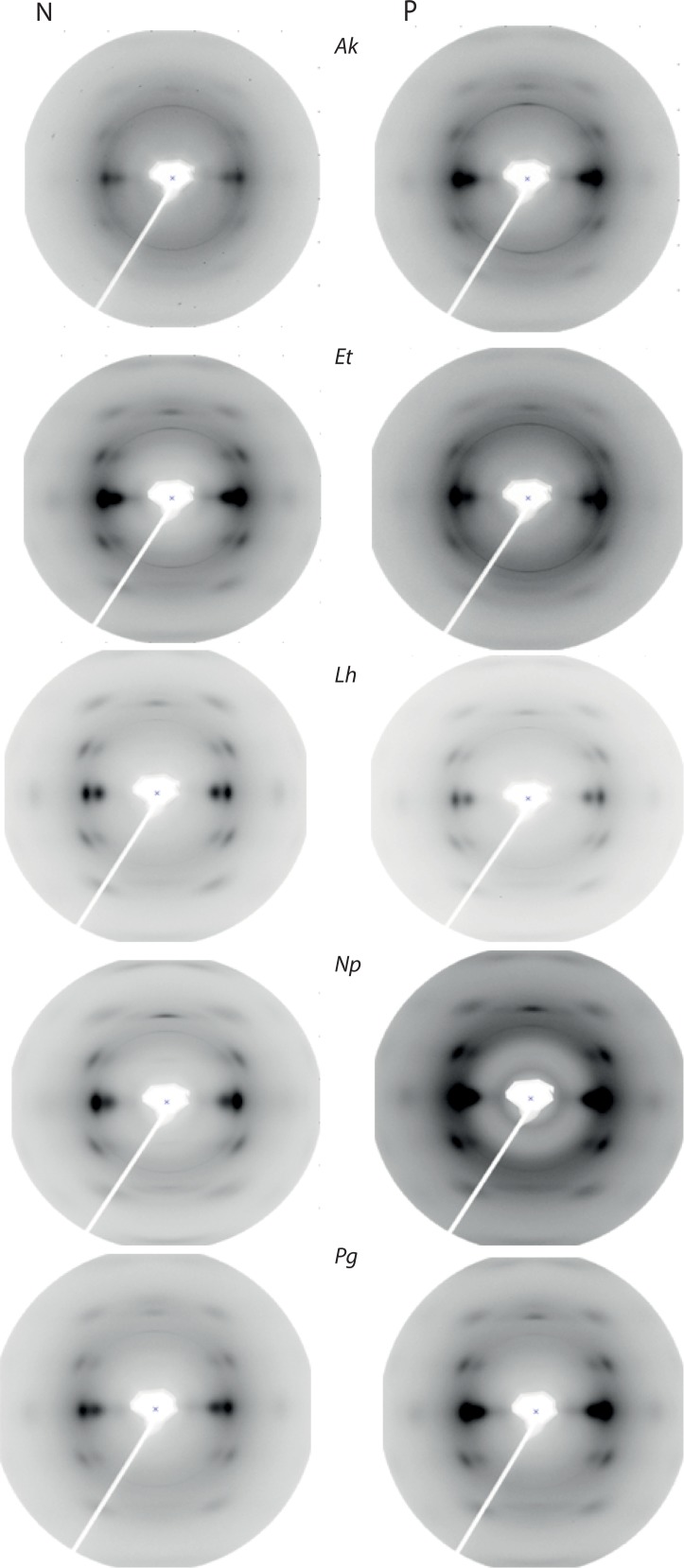
WAXS images derived for MA silks of each species of spider. Where P = protein fed and N = protein deprived treatments, *Ak* = Argiope *keyserlingi*, *Et* = *Eriophora transmarina*, *Lh* = *Latrodectus hasselti Np* = *Nephila plumipes*, *Pg* = *Phonognatha graeffei*.

**Fig 5:**
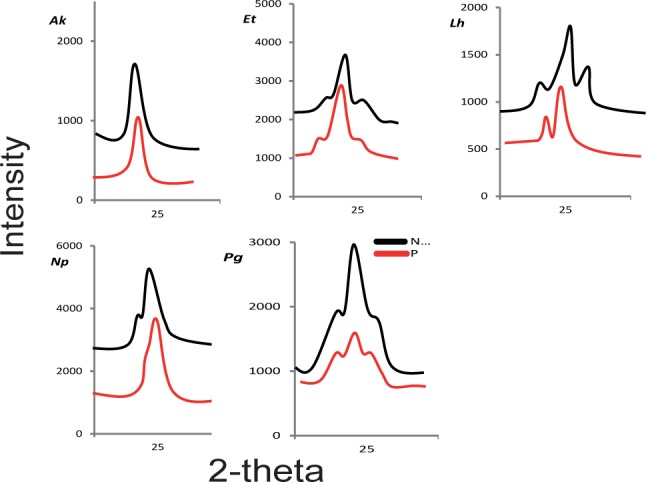
WAXS derived intensity *vs* 2*θ* plots for MA silks of each species. Where P = protein fed and N = protein deprived treatments, *Ak* = Argiope *keyserlingi*, *Et* = *Eriophora transmarina*, *Lh* = *Latrodectus hasselti Np* = *Nephila plumipes*, *Pg* = *Phonognatha graeffei*.

The silk nanostructures varied in response to protein feeding/deprivation among the five spiders (see Supporting Information, S4 Table). The SAXS images and subsequent intensity *vs* scattering vector (*q*) plots for each spider are shown in Fig 2 and Fig 3 respectively. Examples of two dimensional WAXS images are shown in Fig 4 and the subsequent intensity *vs* 2*θ* plots in Fig 5. The azimuthal angles at the (200) and (120) diffraction peaks are in the Supporting Information (see S1 Fig and S2 Fig, respectively). From the various plots we calculated a greater long period in *A*. *keyserlingi* silks when the spiders were protein deprived compared to when they were protein fed. Within species, the nanostructures generally shifted in the same direction as the mechanical properties we predicted them to affect, e.g. long period and/or FWHM of the amorphous halo varied with extensibility in *A*. *keyerlingi* and *N*. *plumipes* (Supporting Information, S4 Table). We thus expect the structural variations to explain much of the variation in mechanical properties.

The FWHM of the (200) peak was greater in *A*. *keyserlingi* silks when protein deprived compared to when protein fed. Crystallinity, on the other hand, was greater in the silks of protein fed *A*. *keyserlingi* (Supporting Information, S4A Table). These findings and the greater long period in the silk of protein deprived *A*. *keyserlingi* indicate that the crystalline nanostructures in their silks were stacked more densely when the spiders were protein fed, and were more aligned and stretched when they were protein deprived. We considered it likely that the greater alignment of the crystalline region proteins in the silks of protein deprived *A*. *keyserlingi* explains the high extensibility of their silks. FWHM at the amorphous halo was greater in the silks of protein deprived compared to protein fed *N*. *plumipes* (S4D Table). This result, and the greater ultimate strength in the silk of protein fed *N*. *plumipes*, indicated that variability in the non-crystalline nanostructures primarily influenced their silk mechanical properties. SAXS/WAXS did not detect any significant nanostructural shifts across treatment for *E*. *transmarina*, *L*. *hasselti* and *P*. *graeffei* silks (Supporting Information, S4B, S4C and S4E Table).

Amino acid compositions of the MA silks varied across treatments and the type of variations found differed for each of the spiders. For instance, we found a reduction in the proline, alanine and glycine compositions of *A*. *keyserlingi* silk when protein deprived (Supporting Information, S5 Table). A reduction in proline composition was, however, detected in *N*. *plumipes* and *P*. *graeffeii* MA silks when they were protein fed.

**Fig 6:**
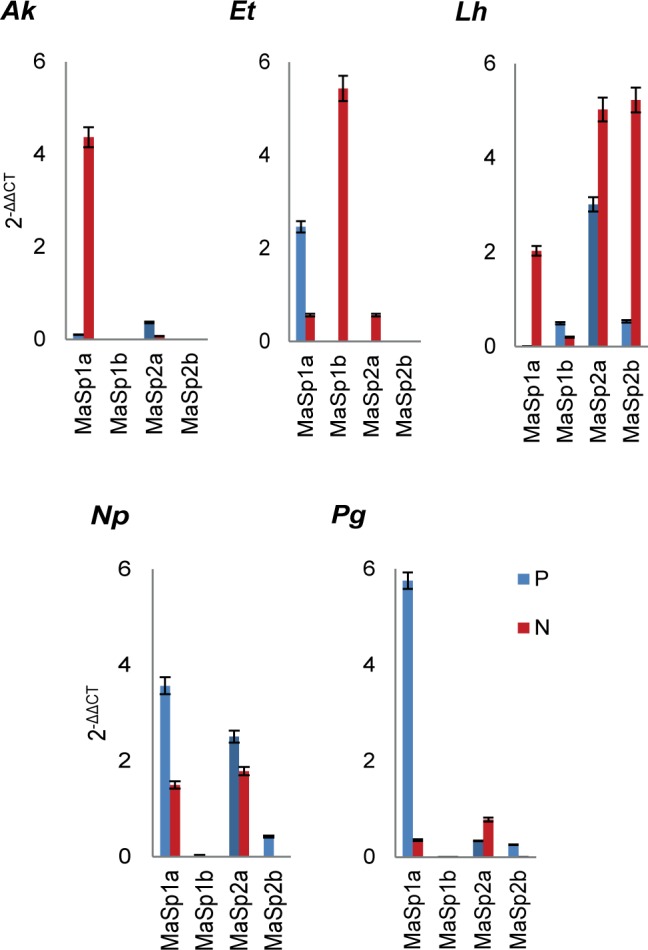
Comparisons of the expressions of the MaSp1 genes previously isolated from the *Argiope trifasciata* (MaSp1a) and *Latrodectus hesperus* (MaSp1b), and the MaSp2 genes from *Argiope trifasciata* (MaSp2a) and *Latrodectus hesperus* (MaSp2b), across treatments for each of the five spiders. Where P = protein fed and N = protein deprived treatments, *Ak* = Argiope *keyserlingi*, *Et* = *Eriophora transmarina*, *Lh* = *Latrodectus hasselti Np* = *Nephila plumipes*, *Pg* = *Phonognatha graeffei*.

Fig 6 shows mean spidroin expression (2^-ΔΔC^_T_) values across treatments for each species. Interestingly, the across treatment expression patterns differed significantly among the five species (Wilk’s λ = 0.011,d.f. = 5,10, P <0.001). We found that *A*. *keyserlingi* upregulated the expression of *MaSp1*a within their MA gland when protein deprived (F_1,28_ = 13.911, p < 0.001). *E*. *transmarina* on the other hand downregulated the *MaSp1*a expression within their MA gland when protein deprived (F_1,28_ = 42.171, p < 0.001), but upregulated *MaSp1*b (F_1,28_ = 8.135, p = 0.005). *L*. *hasselti* upregulated their *MaSp1*a (F_1,28_ = 12.308, p < 0.001), *MaSp2*a (F_1,28_ = 27.604, p < 0.001), and *MaSp2*b expression (F_1,28_ = 25.342, p < 0.001) expressions when protein deprived, while downregulating *MaSp1*b (F_1,28_ = 54.224, p < 0.001), while *N*. *plumipes* (c.f. response to protein deprivation by *N*. *pilipes* [27]) downregulated both their *MaSp1*a (F_1,28_ = 21.104, p < 0.001) and *MaSp2*a (F_1,28_ = 8.358, p = 0.007) expression when protein deprived, which corresponded with an increase in proline composition. *P*. *graeffei* significantly downregulated their *MaSp1*a (F_1,28_ = 6.587, p = 0.010) and upregulated their *MaSp2*a (F_1,28_ = 8.543, p = 0.006) expression when protein deprived.

### What are the contributions of changes in amino acid compositions and nanostructures in inducing spider silk mechanical property variation?

To answer this question we derived additive regression models for three response (Y) variables pertaining to the mechanical properties: ultimate strength, extensibility and toughness, and eight predictor (*x*_*i*_) variables: thread width (*x*_1_), FWHM of the amorphous halo (*x*_2_), FWHM of the (200) diffraction peak (*x*_3_), crystallinity (*x*_4_), long period (*x*_5_), proline composition (*x*_6_), glycine composition (*x*_7_), and alanine composition (*x*_8_) using data from all species excluding *L*. *hasselti*. The variables chosen were those that our analyses above found to differ across treatments in at least one species. Our models showed crystallinity to predominantly influence the ultimate strength in nutritionally affected MA silks (Table 2A). Long period, which represents the nanostructure alignment across the silk’s amorphous and lamellar regions, and alanine and proline composition, were predominantly influential over extensibility (Table 2B). Long period, along with glycine, alanine and proline compositions, influenced silk toughness (Table 2C).

**Table 2:** Results of additive regression models for three response (Y) variables: the mechanical properties ultimate strength (model a), extensibility (model b) and toughness (model c), and eight predictor (*x*_*i*_) variables: thread width (*x*_1_), FWHM of the amorphous halo (*x*_2_), FWHM of the (200) diffraction peak (*x*_3_), crystallinity (*x*_4_), long period (*x*_5_), proline composition (*x*_6_), glycine composition (*x*_7_), and alanine composition n (*x*_8_). These parameters were used because species-specific MANOVAs found them to significantly differ across treatments. Data from all five species studied were pooled to construct the models.

(a) Ultimate strength	Predictor variable	b estimate	Standard error of b	Standard co-efficient	t_1_	P-value
	Intercept	10829	3517.103	0	3.070	0.054
	thread width (*x*_1_)	2.448	1.877	0.030	1.305	0.283
	FWHM: amorphous halo (*x*_2_),	89.601	0.971	-0.375	-2.622	0.078
	FWHM: (200) (*x*_3_)	-368.230	1.874	-0.272	-1.214	0.311
	X_c_ (*x*_4_)	2.761	1.001	0.158	3.756	0.040*
	*L* (*x*_5_)	-14.814	4.081	-0.056	-3.031	0.056
	% proline (*x*_6_)	57.631	35.095	0.631	1.639	0.348
	% glycine (*x*_7_)	0.880	29.037	0.023	0.230	0.081
	% alanine (*x*_8_)	53.030	24.886	0.289	2.157	0.176
(b) Extensibility	Predictor variable	b estimate	Standard error of b	Standard co-efficient	t_1_	P-value
	Intercept	0.656	0.388	0	1.771	0.327
	thread width (*x*_1_)	-0.061	0.027	-0.696	-2.273	0.163
	FWHM: amorphous halo (*x*_2_),	0.159	0.058	0.395	2.720	0.104
	FWHM: (200) (*x*_3_)	-0.188	0.038	-0.031	-1.214	0.429
	X_c_ (*x*_4_)	0.154	0.019	0.132	1.167	0.342
	*L* (*x*_5_)	0.018	0.005	0.315	3.784	0.020*
	% proline (*x*_6_)	0.071	0.014	0.472	4.089	0.012*
	% glycine (*x*_7_)	0.026	0.011	0.360	2.218	0.065
	% alanine (*x*_8_)	-0.044	0.010	0.502	-3.354	0.043*
(c) Toughness	Predictor variable	b estimate	Standard error of b	Standard co-efficient	t_1_	P-value
	Intercept	-80.046	44.049	0	-1.817	0.120
	thread width (*x*_1_)	-27.372	3.041	0.135	-2.447	0.067
	FWHM: amorphous halo (*x*_2_),	68.411	6.639	0.168	2.304	0.061
	FWHM: (200) (*x*_3_)	-12.316	3.499	0.080	-1.519	0.176
	X_c_ (*x*_4_)	25.414	10.463	0.056	2.248	0.084
	*L* (*x*_5_)	4.259	0.671	0.134	5.351	0.009*
	% proline (*x*_6_)	23.488	1.640	0.201	7.312	<0.001*
	% glycine (*x*_7_)	14.651	1.630	0.153	5.853	0.005*
	% alanine (*x*_8_)	-12.591	1.517	0.124	-3.924	0.015

* denotes the significantly influential predictor variable(s) for each model.

## Discussion

Our study was the first to comprehensively examine the consequences of protein deprivation on variations in spider silk mechanics, structures, amino acid compositions and gene expressions at multiple scales across species. We concluded that: (i) MA silk properties of the spiders respond differently across multiple scales to variations in nutritional intake, and (ii) variations in spidroin expression and the crystalline and non-crystalline nanostructures play specific roles in inducing variations in the silk’s mechanical properties.

The *MaSp1*: *MaSp2* expression patterns we found across treatments generally did not correspond with the amino acid compositions according to our expectations under a traditional *MaSp* expression model. For instance, a decreased in alanine and glycine composition in the MA silk of protein deprived *A*. *keyserlingi* was not associated with any form of *MaSp*2 downregulation, but was associated with an upregulation of *MaSp1*a. The spidroin expression pattern found for *E*. *transmarina* across treatments likely explains why the amino acid compositions did not vary across treatments in this spider. The high expression of all of the *MaSp1* and *MaSp2* paralogs in the MA glands of *L*. *hasselti* is consistent with findings for *L*. *hasperus* [24,68,74]. Expression of multiple *MaSp* paralogs in the MA glands was not common for most species we assessed, but was pronounced in *L*. *hasselti*. The increase in proline composition in protein deprived *N*. *plumipes* MA silk conflicts with the findings of Blamires et al. [27], who found an increase in proline composition in protein deprive *N*. *pilipes*. We would have expected the downregulation of *MaSp2*a expression by this spider when protein deprived to result in a decrease in silk proline composition. The downregulation of *MaSp1*a on the other hand was unlikely to have any influence on proline composition [51,75,76]. An increase in the proline composition in the silks of protein deprived *P*. *graefei*, however, may be ascribed to an increased in *MaSp2*a expression by these spiders.

The apparent disconnect between the spidroin expression patterns and the amino acid compositions in the silks of most species might lead us to conclude that: (1) the silk nanostructures and mechanical properties of different spiders do not respond similarly to variations in spidroin expression, and (2) the relative contributions of change in spidroin expression in inducing spider silk mechanical property variation is minimal at best. We, nevertheless, presume caution before drawing definitive conclusions about the influence of *MaSp* expression in light of the following. Firstly, in our RT-PCR reactions any specific amplification of *MaSp* paralogs could arise in any given species using a single pair of primers [65]. This may cause falsely elevate expression levels of certain *MaSp* transcripts. Examination of the melt curves for the genes screened suggested that in some species there were indeed multiple amplifications. The most discernible example of this is found in the melt curves for *MaSp1*a and *MaSp2*a, particularly for *Nephila plumipes* (see S3 Fig). Secondly, the amplification of non-orthologous *MaSp* loci across species with single primer pairs could lead to falsely elevated or falsely lowered detection of expression levels [77]. Any of the *MaSp*-targeting primers could thus have identified a single *MaSp* transcript in one species, a single non-orthologous transcript in a second species, and/or multiple non-orthologous transcripts in yet another species. Thirdly, since the full length *MaSp* sequences for each of the species under investigation herein is not known, we designed primers based on the *MaSp* sequences for closely related species (*Argiope trifasciata* and *Latrodectus hesperus*). The amplification efficiency thus may potentially be biased toward species with the greatest sequence homology (i.e. *Argiope keyserlingi* and *Latrodectus hasselti*). Indeed only in *L*. *hasselti* was expression of *MaSp1*b and *MaSp2*b comparable to that of *MaSp1*a and *MaSp2*a. Lastly, some degree of gDNA amplification cannot be ruled out as influencing our results. We, however, did not expect this to cause major expression value biases within any particular species.

While the above caveats may tempt us to think that the RT-PCR analyses yielded largely uncertain results, we found that the expression patterns for *P*. *graeffei* aligned exceptionally close to our expectation should the spiders be regulating *MaSp*1 and *MaSp*2 expression alone. We expect that such a finding would not have been possible if our primer choices and/or amplifications were compromised in any way. Moreover, our expression values conformed with those reported for the *MaSp*-a, *MaSp*-f, and *MaSp*-g genes by Babb et al. [10] (although these authors reported a wider range of values for other *MaSp* loci). We thus expect that the across-treatment expression patterns that we reported for each species to be reliable for the explicit purpose of checking *MaSp* expression against the amino acid compositions for each species.

Our study corroborates work showing that amino acid compositional shifts confer consequences on crystalline and non-crystalline protein structures [11,17,26,27,49] and alignments within the crystalline, amorphous and lamellar regions of the silk, which in turn influences its strength, extensibility and toughness [1,40,43,50,52]. Our study also uncovered novel mechanisms behind the multilevel shifts in silk properties, as follows.

While silk mechanical properties were affected by protein deprivation in four of the five species of spider (the exception being *L*. *hasselti*), the mechanisms by which the mechanics were affected differed in each instance. Our amino acid composition analyses, for instance, found a lowering of proline, alanine and glycine compositions in the silk of *A*. *keyserlingi* when protein deprived. Nevertheless the silks of *N*. *plumipes* and *P*. *graeffei* had greater proline compositions when protein deprived. Our genetic expression analyses revealed that most spiders seemed to preferentially regulate their expression of the *MaSp1* genes rather than *MaSp2* genes across treatments, contrary to what might be predicted, given the MaSp2 protein is expected to be the more costly of the two proteins to metabolically synthesize [27,51,75].

Our SAXS measurements revealed that the spidroin regulation and consequent variability in silk proline corresponded with variations in long period (*L*) in *A*. *keyserlingi*, *N*. *plumipes* and *P*. *graeffei*, suggesting that spidroin expression and/or proline composition affects nanostructural alignment in the amorphous and lamellar regions. These compositional and structural variations correlated well with extensibility and toughness variations in the silks, providing first evidence of a functional link between spidroin expression, nanostructural formation, and mechanical property variations in the MA silks of different spiders.

An explanation for the association between proline and amorphous region nanostructural alignment might lie in the capacity for proline to form cross-linkages which disrupt the hydrogen bonds between amorphous region α-helices and other structures causing slippage in the nanostructures under strain [20]. Our finding of a lack of any change in amorphous and lamella region alignment in the silks of protein deprived *P*. *graeffei*, despite significant variations in proline composition, across treatments nonetheless suggests that proline does not necessarily directly affect amorphous region nanostructural alignment but likely provides the conditions for the breaking and re-establishment of hydrogen bonds in the region [78].

Our amino acid composition and WAXS analyses, and subsequent modelling, predicted that, in contrast to extensibility and toughness, MA silk ultimate strength was primarily influenced by variations in crystallinity independent of *MaSp1*: *MaSp2* expression or any subsequent shifts in amino acid composition. Our WAXS analyses withal suggested that variations in amorphous region nanostructural alignment and crystallinity combine to influence silk strength. Glandular pH, salts and shear stresses during spinning influence the formation of the crystalline nanostructures [11]. Hence, we deduced that between treatment variations in glandular pH, salts and shear stress induce the crystalline region proteins to undergo α-helix→β-sheet nanostructural phase transitions within the spinning duct [79]. These transitions result in an increase in crystalline density [50], which causes the crystalline region nanostructures to realign under strain [1]. Such an increase in crystalline density should be identifiable in WAXS experiments as a reduction in 2θ and FWHM at the (200) or (120) diffraction peaks [32]. Indeed, our analyses herein revealed such a mechanism occurred in protein deprived *N*. *plumipes* silks (S1 Table). Nonetheless, the phenomenon was not detected in the silks of any other species, leading us to conclude that the precise physiological mechanisms inducing property variation differed among the five species.

Unlike the other four species, we found that neither protein feeding nor deprivation effected the amino acid compositions, nanostructures, or mechanical properties of *L*. *hasselti*’s MA silk. A similar lack of variability in MA silk structure and mechanical properties was found in *L*. *hasselti* collected at different times of year [26]. Spiders of the genus *Latrodectus* use MA silk within their three-dimensional cobwebs as structural supports [80], whereas the orb web building spiders use MA silks within webs to absorb the impact of flying prey [11]. A testable hypothesis might accordingly be that orb web spider MA silks have a greater inherent variability in order to adjust the functionality of the orb web [81] and this variability is triggered by changes in nutrient uptake. The MA silks of cobweb spiders on the other hand do not require such property variability so are not so sensitive to changes in nutrient uptake. Our gene expression and amino acid composition analyses alluded to the possibility of the expression of multiple spidroins in *L*. *hasselti*, as has been found in *L*. *hesperus* [24,68,72,74]. The differential expression of a multitude of spidroins might thus be a mechanism by which cobweb building spiders maintain silk property homeostasis across variable nutritional environments.

In summary, we found that the MA silk properties of five species of Araneoid spiders varied in response to similar variations in protein intake. Stronger and tougher silks with greater crystallinity and amorphous region nanostructural alignments were found for protein fed *P*. *graeffei* and *N*. *plumipes* which contrasted with the findings for *A*. *keyserlingi* and those reported previously for protein fed/deprived *N*. *pilipes* [27]. The properties and nanostructures of *L*. *hasselti*’s MA silks were unaffected by nutrient deprivation. Our analyses suggested that variations in *MaSp1*: *MaSp2* expression were largely ineffectual over amino acid compositions. Proline and alanine composition and the crystalline and amorphous nanostructures significantly varied in all species with the exception of *Latrodectus hasselti*, all with subsequent effects on mechanical properties. We uncovered additional unexpected and novel findings regarding the mechanisms inducing variations at different levels in different spiders. For instance, *MaSp2* genes were not as strongly regulated as we might have predicted under the current *MaSp* model when protein intake changed in *A*. *keyserlingi*, *N*. *plumipes* or *P*. *graeffei*. Rather *MaSp1* was more likely to be up or downregulated in these spiders. Our modelling showed that variations in silk strength were associated with variations in crystallinity and the size, length and alignment of the crystalline and non-crystalline proteins independent of expressions of the *MaSp* genes. Extensibility and toughness on the other hand were driven by variations in the crystalline, amorphous and lamellar region nanostructural alignments, which were largely disassociated from *MaSp* expression.

## Conclusions

Here we holistically examined the consequences of gene expression on silk proteins and protein structure and, ultimately, silk functional properties and established that: (1) the MA silk properties of five species of Araneoid spiders varied differently in response to similar variations in protein intake, and (2) the roles of spidroin expression, crystalline, and amorphous region nanostructures on mechanical property variations differed across the species examined.

While there is broad micro-scale homogeneity in the MA silks of Araneoid spiders, our measurements found that variations in the gene expression, amino acid compositions, and nanostructures, further induce mechanical property variations between and within species. Our modelling found nanostructural variations to primarily influence silk extensibility and toughness while variations in the alignment of the crystalline and non-crystalline proteins influenced ultimate strength independent of *MaSp* expressions. Our study provides insights into the nanoscale mechanisms of nutritionally induced spider silk property variability by showing how spidroin expression and nanostructures affect spider silk mechanical property variations in different species. These insights further our understanding of MA silk property variability at multiple levels which is imperative if materials that match the performance of naturally spun spider silks are to one day be synthesized.

## Supporting information

S1 Table Primers used for the RT-PCR analyses, and their genbank accession numbers and sequences. (DOCX)

S2 Table Normalized threshold cycle (C_T_) values for each of the four genes screened from the major ampullate silk glands of each of the five species (*Argiope keyserlingi*, *Eriophora transmarina*, *Latrodectus hasselti*, *Nephila plumipes* and *Phonognatha graeffei*) across the protein fed and protein deprived treatments. (DOCX)

S3 Table Means (±SE) mechanical property values. Contains statistics for five (one per species) single-factor multivariate analyses of variance.
(DOCX)

S4 Table Means (±SE) nanostructure parameter values. Contains statistics for five (one per species) single-factor multivariate analyses of variance.
(DOCX)

S5 Table Means (±SE) amino acid composition values. Contains statistics for five (one per species) single-factor multivariate analyses of variance.
(DOCX)

S1 Fig Examples of WAXS derived MA silk intensity *vs* azimuthal angle plots at the (200) diffraction peaks. (DOCX)

S2 Fig Examples of WAXS derived MA silk intensity *vs* azimuthal angle plots at the (120) diffraction peaks. (DOCX)

S3 Fig Melt curves for MaSp1a and MaSp2a for the five species. Includes both protein deprived and protein fed spiders.
(DOCX)
