# Supplementary material for: Multiscale mechanisms of nutritionally induced property variation in spider silks
Source: PLoS One. 2018 Feb 1;13(2):e0192005. doi: 10.1371/journal.pone.0192005 (PMC5794138; doi:10.1371/journal.pone.0192005)
Supplement: S1 Table — (DOCX) [file pone.0192005.s001.docx]

**S1 Table.** Primers used for the RT-PCR analyses, and their genbank accession numbers and sequences.

| Gene | Genbank accession# | Primer sequence | Description | Reference |
| --- | --- | --- | --- | --- |
| MaSp1a | AF350266.1 | GCAGCTGCAGCCGCAGCAGCAGCAGCCGGTGGCCAAGGAGGACAAGGTGGATATGACGGATTAGGTTCTCAAGGAGCCGGTCAAGGAGGATACGGACAAGGAGGAGCCGCTGCCGCAGCAGCCGCAGCCAGTGGAGCTGGTAGTGCCCAACGAGGAGGCTTAGGTGCTGGAGGTGCAGGACAAGGATATGGAGCCGGATCAGGCGGTCAAGGAGGAGCTGGACAAGGTGGCGCAGCTGCAGCCACAGCAGCAGCAGCCGGTGGCCAAGGAGGACAAGGTGGATATGGCGGATTAGGTTCCCAAGGATCCGGTCAAGGAGGATACGGACAAGGAGGAGCCGCTGCCGCAGCAGCCGCAGCCAGTGGAGATGGTGGTGCCGGACAAGAAGGCTTAGGTGCTGGAGGTGCAGGACAAGGATATGGTGCTGGATTAGGCGGTCAAGGAGGAGCTGGACAAGGTGGCGCAGCTGCAGCCGCAGCAGCAGCAGCCGGTGGCCAAGGAGGACAAGGTGGATATGGCGGATTAGGTTCTCAAGGAGCCGGTCAAGGAGGATACGGACAAGGAGGAGCCGCTGCCGCAGCAGCCGCAGCCAGTGGAGCTGGTGGCGCCGGACAAGGAGGCTTAGGTGCTGCAGGTGCAGGACAAGGATATGGTGCCGGATCAGGCGGTCAAGGAGGAGCTGGACAAGGTGGCGCAGCTGCAGCTGCAGCAGCAGCAGCCGGTGGCCAAGGAGGACAAGGTGGATATGGCGGATTAGGTTCTCAAGGAGCCGGTCAAGGAGGATACGGACAAGGAGGAGTCGCTGCTGCAGCAGCCGCAGCCAGTGGAGCTGGTGGTGCCGGACGAGGAGGCTTAGGTGCTGGAGGTGCAGGACAAGAATATGGTGCCGTATCAGGCGGTCAAGGAGGAGCTGGACAAGGTGGCGAAGCTGCAGCCGCAGCAGCAGCAGCCGGTGGCCAAGGAGGACAAGGTGGATATGGCGGATTAGGTTCTCAAGGAG | *Argiope trifasciata* major ampullate spidroin 1 (MaSp1) mRNA, partial cds | Gatesy et al. (2001) |
| MaSp1b | AY953074.1 | TGGTTATGGAGGACAAGGTGCCGGACAAGGTGGAGCTGGAGCTGCAGCCGCAGCAGCAGCTGCTGGAGGTGCAGGTCAAGGAGGACAAGGTGGATATGGACAAGGAGGATACGGACAAGGTG | *Latrodectus hesperus* isolate LT51 major ampullate spidroin 1 mRNA,partial cds | Garb and Hayashi (2005) |
| MaSp2a | AF350267.1 | CGCTGGACCAGGATACGGACCAGGAGCCGGACAACAAGGACCTGGAAGTCAAGGACCAGGAAGTGGTGGACAACAAGGACCTGGTGGACAAGGACCATATGGACCAAGCGCTGCCGCCGCAGCAGCTGCCGCTGGACCAGGATATGGACCAGGAGCTGGACAACAAGGACCAGGAAGTGGCGGACAACAAGGAGGCCAAGGATCTGGACAGCAAGGACCAGGAGGTGCCGGTCAAGGAGGTCCTCGTGGTCAAGGACCATACGGACCAGGTGCAGCCGCCGCCGCCGCAGCTGCTGGAGGATACGGACCAGGAGCTGGACAACAAGGACCTGGAAGTCAAGGACCCGGAAGTGGTGGACAACAAGGTCCTGGTAGTCAAGGACCATATGGACCAAGTGCAGCCGCAGCAGCAGCAGCCGCTGGACCAGGATACGGACCAGGAGCCGGACAACAAGGACCTGGAAGTCAAGGACCAGGAAGTGGTGGACAACAAGGACCTGGTGGACAAGGACCATATGGACCAAGCGATGCCGCCGCAGCAGCTGCCGCTGGACCAGGATATGGACCAGGAGCTGGACAACAAGGACCAGGAAGTGGCGGACAACAAGGAGGCCAAGGATCTGGACAGCAAGGACCAGGAGGTGCCGGTCAAGGAGGTCCTCGTGGTCAAGGACCATACGGACCAGGTGCAGCCGCCGCCGCCGCAGCTGCTGGAGGATACGGACCAGGAGCTGGACAACAAGGACCTGGAAGTCAAGGACCCGGAAGTGGTGGACAACAAGGTCCTGGTAGTCAAGGACCATATGGGCCAAGTGCAGCCGCAGCAGCAGCAGCCGCTGGACCAGGATACGGACCAGGAGCCGGACAACAAGGACCTGGAAGTCAAGGACCAGGAAGTGGTGGACAACAAGGTCCTGGTAGTCAAGGACCATATGGACCAAGTGCAGCCGCAGCAGCAGCAGCCGCTGGACCAGGATACGGACCAGGAGCCGGACAACAA | *Argiope trifasciata* major ampullate spidroin 2 (MaSp2) mRNA, partial cds | Gatesy et al. (2001) |
| MaSp2b | AY953075.1 | CAACTAATGCGGCAGCTCTTTCTAATGTCATTAGTAATGCCGTTTCCCAAGTCAGTGCAAGTAATCCAGGATCTTCCTCTTGTGATGTCCTTGTTCAAGCACTTCTTGAAATAATTACTGCATTAATTAGTATACTAGATTCCTCTAGTGTTGGACAAGTTAATTACGGTTCTTCAGGACAGTATGCACAAATTGTAGGGCAGTCTATGCAACAAGCTATGGGGT | *Latrodectus hesperus* isolate LT57 major ampullate spidroin 2 mRNA,partial cds. | Garb and Hayashi (2005) |
| House-keeping1 | DQ363216.1 | TCCATTCCAATATGCAAATGCTGTTTCTAACGCGTTTGGACAGTTACTGGGAGAACAAGGAATTTTAACACAAGAAAATGCTTCCTCTTTAGCCTCTTCAGTTGCTAATGCTTTATCTGCATCTTCTTCATTAGTTCCTTCTGCTATTAGCACAGGTGTTCCTGGTTTGATTGTAGGACCCTCTATTGTTTCTTCATTGAATGCTCCGATTGCAGGATTTGCTGTTCCTGGAGTAGCGCAAGTGATTGTACCAACTGCATATTCCACACTTTTAGCACCAGTACTTTCACCTGCTGGATTGGCTTCTACTGCAGCAACTTCAAGAATTAATGACATTGCACAAAGTTTATCTTCAACTCTATCTTCAGGATCGCAATTAGCTCCAGATAATGTACTTCCTGGTCTCATTCAACTGTCTTCATCCATTCAAAGCGGAAATCCTGATTTAGACCCTGCTGGTGTTTTGATCGAGTCATTATTAGAATACACTTCCGCACTTTTAGCTCTTCTTCAAAACGCTCAAATTACAACTTATGATGCTGCGACTTTACCTGCATTCAGTACAGCTCTTGTAAATTACCTTGTTCCCCTTGTTTA | *Drosophila rufa* g3pdh, partial cds. |  |
| House-keeping2 | AY426339 | GTGGATATGGAGGTCTTGGTGGACAAGGTGCCGGACAAGGAGCTGGTGCAGCCGCCGCAGCAGCAGCTGGTGGTGCCGGACAAGGAGGATATGGAGGTCTTGGAAGCCAAGGTGCTGGACGAGGTGGACAAGGTGCAGGCGCAGCCGCAGCCGCAGCTGGAGGTGCTGGTCAAGGAGGATACGGAGGTCTTGGAAGCCAAGGTGCTGGACGAGGAGGATTAGGTGGACAAGGTGCAGGTGCAGCAGCAGCAGCTGGAGGTGTCGGACAAGGAGGACTAGGTGGACAAGGTGCTGGACAAGGAGCTGGAGCAGCTGCTGCAGCAGCTGGTGGTGCCGGACAAGGAGGATATGGAGGTCTCGGAAGCCAAGGTGCAGGACGAGGTGGATCAGGTGGACAAGGGGCAGGTGCAGCAGCAGCAGCAGCTGGAGGTGCCGGACAAGGAGGATATGGAGGTCTTGGAAGCCAAGGTGCAGGACGAGGTGGATTAGGTGGACAGGGTGCAGGTGCAGCAGCAGCAGCAGCAGCCGGAGGTGCTGGACAAGGAGGATACGGTGGTCTTGGTGGACAAGGTGCCGGACAAGGTGGCTATGGAGGACTTGGAAGCCAAGGTGCTGGACGAGGAGGATTAGGTGGACAAGGTGCAGGTGCAGCAGCAGCAGCTGGAGGTGCCGGACAAGGAGGACTAGGTGGACAAGGAGCTGGAGCAGCCGCTGCAGCAGCTGGTGGTGCCGGACAAGGAGGATATGGAGGTCTTGGAAGCCAAGGTGCTGGACGAGGTGGACAAGGTGCAGGCGCAGCCGCAGCAGCAGCCGGAGGTGCTGGACAAGGAGGATACGGTGGACAAGGTGCCGGACAAGGAGGCTATGGAGGACTTGGAAGCCAAGGTGCTGGACGAGGAGGATTAGGTGGACAAGGTGCAGGTGCAGCAGCAGCAGCAGCAGCAGCTGGAGGTGCCGGACAAGGAGGATTAGGTGGACAAGGTGCAGGTGCAGCAGCAGC | *Argiope trifasciata* aciniform spidroin 1, mRNA, partial cds | Hayashi et al. (2004) |
