# Supplementary material for: Multiscale mechanisms of nutritionally induced property variation in spider silks
Source: PLoS One. 2018 Feb 1;13(2):e0192005. doi: 10.1371/journal.pone.0192005 (PMC5794138; doi:10.1371/journal.pone.0192005)
Supplement: S2 Table — (DOCX) [file pone.0192005.s002.docx]

**S2 Table.** Normalized threshold cycle (C_T_) values for each of the four genes screened from the major ampullate silk glands of each of the five species (*Argiope keyserlingi*, *Eriophora transmarina*, *Latrodectus hasselti*, *Nephila plumipes* and *Phonognatha graeffei*) across the protein fed and protein deprived treatments.

|  |  | C_T_ value | |
| --- | --- | --- | --- |
|  | Gene | Protein deprived | Protein fed |
| *Argiope keyserlingi* | MaSp1a | 9.442 | 18.194 |
|  | MaSp1b | 0 | 0 |
|  | MaSp2a | 4.374 | 6.808 |
|  | MaSp2b | 0 | 0 |
|  | Housekeeping | 9.988 | 8.753 |
|  |  |  |  |
| *Eriophora transmarina* | MaSp1a | 9.324 | 24.285 |
|  | MaSp1b | 15.753 | 11.384 |
|  | MaSp2a | 1.0 | 0.942 |
|  | MaSp2b | 0 | 0 |
|  | Housekeeping | 2.422 | 1.591 |
|  |  |  |  |
| *Latrodectus hasselti* | MaSp1a | 6.54 | 4.006 |
|  | MaSp1b | 8.991 | 22.446 |
|  | MaSp2a | 6.162 | 3.791 |
|  | MaSp2b | 9.007 | 21.433 |
|  | Housekeeping | 24.113 | 22.544 |
|  |  |  |  |
| *Nephila plumipes* | MaSp1a | 8.65 | 20.285 |
|  | MaSp1b | 7.107 | 0 |
|  | MaSp2a | 8.551 | 18.246 |
|  | MaSp2b | 0 | 2.0 |
|  | Housekeeping | 9.181 | 9.381 |
|  |  |  |  |
| *Phonognatha graeffei* | MaSp1a | 11.171 | 23.1713 |
|  | MaSp1b | 9.645 | 9.181 |
|  | MaSp2a | 10.714 | 12.629 |
|  | MaSp2b | 0 | 8.912 |
|  | Housekeeping | 1.388 | 0.941 |
|  |  |  |  |
