# Supplementary material for: Multiscale mechanisms of nutritionally induced property variation in spider silks
Source: PLoS One. 2018 Feb 1;13(2):e0192005. doi: 10.1371/journal.pone.0192005 (PMC5794138; doi:10.1371/journal.pone.0192005)
Supplement: S3 Table — Contains statistics for five (one per species) single-factor multivariate analyses of variance. (DOCX) [file pone.0192005.s003.docx]

**S3 Table.** Means (±S.E) thread widths and values for the mechanical properties: (i) ultimate strength, (ii) extensibility, (iii) Young’s modulus, and (iv) toughness, across the protein fed and protein deprived feeding treatments. Adjusted R^2^ statistics for a sum-of-squares (SS) for the whole model against SS for the residuals model are shown as estimates of the proportion of variance explained. Statistics for the five (one per species) single-factor multivariate analyses of variance (Wilk’s λ, with * denoting significance variations across treatments at α < 0.05) are shown, as are P-values are for Fisher’s Least Significant Difference tests (with * denoting significance differences between treatments).

|  |  | Treatment (means ±SE) | | Adjusted R^2 (^SS Whole vs SS Residuals) | Wilk’s λ (d.f. = 5,10) | Fisher’s P-value |
| --- | --- | --- | --- | --- | --- | --- |
| (a) *Argiope keyserlingi* | parameters | Protein deprived | Protein fed |  | 0.022* |  |
|  | Thread width | 3.39 ± 0.18 | 2.17 ± 0.15 | 0.530 |  | <0.001* |
|  | Ultimate strength (MPa) | 290.774 ± 36.403 | 458.048 ± 97.187 | 0.187 |  | 0.132 |
|  | Extensibility (mm/mm) | 0.569 ± 0.023 | 0.471 ± 0.052 | 0.232 |  | 0.008* |
|  | Young’s Modulus (GPa) | 8.608 ± 1.051 | 10.739 ± 0.754 | 0.102 |  | 0.122 |
|  | Toughness (MJ/m^3^) | 89.199 ± 14.174 | 94.229 ± 26.932 | 0.044 |  | 0.870 |
| (b) *Eriophora transmarina* |  |  |  |  | 0.010* |  |
|  | Thread width | 5.83 ± 0.46 | 4.24 ± 0.47 | 0.128 |  | 0.095 |
|  | Ultimate strength (MPa) | 392.031 ± 61.994 | 327.887 ± 47.977 | 0.020 |  | 0.416 |
|  | Extensibility (mm/mm) | 0.354 ± 0.063 | 0.504 ± 0.062 | 0.318 |  | 0.023* |
|  | Young’s Modulus (GPa) | 8.777 ± 1.247 | 11.566 ± 0.916 | 0.129 |  | 0.094 |
|  | Toughness (MJ/m^3^) | 85.128 ± 19.406 | 115.724 ± 28.009 | 0.074 |  | 0.060 |
| (c) *Latrodectus hasselti* |  |  |  |  | 0.008 |  |
|  | Thread width | 3.10 ± 0.161 | 3.01 ± 0.15 | 0.058 |  | 0.676 |
|  | Ultimate strength (MPa) | 399.942 ± 97.299 | 260.803 ± 52.744 | 0.037 |  | 0.231 |
|  | Extensibility (mm/mm) | 0.315 ± 0.067 | 0.353 ± 0.044 | 0.027 |  | 0.425 |
|  | Young’s Modulus (GPa) | 10.578 ± 1.449 | 10.155 ± 1.068 | 0.063 |  | 0.743 |
|  | Toughness (MJ/m^3^) | 120.185 ± 22.262 | 97.044 ± 15.953 | 0.090 |  | 0.107 |
| (d) *Nephila plumipes* |  |  |  |  | 0.017* |  |
|  | Thread width | 3.37 ± 0.22 | 3.73 ± 0.09 | 0.083 |  | 0.146 |
|  | Ultimate strength (MPa) | 595.945 ± 145.658 | 433.284 ± 98.481 | 0.262 |  | 0.027* |
|  | Extensibility (mm/mm) | 0.369 ± 0.043 | 0.413 ± 0.045 | 0.033 |  | 0.434 |
|  | Young’s Modulus (GPa) | 7.983 ± 0.933 | 9.476 ± 1.510 | 0.019 |  | 0.414 |
|  | Toughness (MJ/m^3^) | 157.118 ± 35.248 | 127.475 ± 32.021 | 0.168 |  | 0.031* |
| (e) *Phonognatha graeffei* |  |  |  |  | 0.015* |  |
|  | Thread width | 3.15 ± 0.214 | 3.13 ± 0.29 | 0.071 |  | 0.597 |
|  | Ultimate strength (MPa) | 472.754 ± 93.289 | 384.115 ± 16.361 | 0.267 |  | 0.025* |
|  | Extensibility (mm/mm) | 0.316 ± 0.043 | 0.326 ± 0.027 | 0.069 |  | 0.757 |
|  | Young’s Modulus (GPa) | 9.971 ± 1.072 | 6.293 ± 1.382 | 0.176 |  | 0.065 |
|  | Toughness (MJ/m^3^) | 77.734 ± 13.973 | 59.581 ± 5.492 | 0.287 |  | 0.023* |
