# Supplementary material for: Multiscale mechanisms of nutritionally induced property variation in spider silks
Source: PLoS One. 2018 Feb 1;13(2):e0192005. doi: 10.1371/journal.pone.0192005 (PMC5794138; doi:10.1371/journal.pone.0192005)
Supplement: S4 Table — Contains statistics for five (one per species) single-factor multivariate analyses of variance. (DOCX) [file pone.0192005.s004.docx]

**S4 Table.** Means (±S.E) values for the nanostructure parameters (i) long period (*L*), (ii) 2*θ* FWHM of the (200) Bragg diffraction peak, (iii) 2*θ* FWHM of the (120) Bragg diffraction peak, (iv) 2*θ* FWHM of the amorphous region, (v) relative crystalline intensity ratios (*I*_200_/ *I*_120_), (vi) crystallinity index (X_c_), and Herman’s orientation function (vii) (f_c_,), across the protein fed and protein deprived feeding treatments. Adjusted R^2^ statistics for a sum-of-squares (SS) for the whole model against SS for the residuals model are shown as estimates of the proportion of variance explained. Statistics for the five (one per species) single-factor multivariate analyses of variance (Wilk’s λ, with * denoting significance variations across treatments at α < 0.05) are shown, as are P-values are for Fisher’s Least Significant Difference tests (with * denoting significance differences between treatments).

|  |  | Treatment (means ±SE) | | Adjusted R^2 (^SS Whole vs SS Residual) | Wilk’s λ (d.f. = 5,10) | Fisher’s P-value |
| --- | --- | --- | --- | --- | --- | --- |
| (a) *Argiope keyserlingi* | parameters | Protein deprived | Protein fed |  | 0.030* |  |
|  | *L* | 19.266 ± 0.354 | 13.195 ± 0.294 | 0.569 |  | <0.001* |
|  | 2*θ* FWHM (200) | 3.263 ± 0.121 | 4.265 ± 0.586 | 0.383 |  | 0.005* |
|  | 2*θ* FWHM (120) | 1.784 ± 0.020 | 1.686 ± 0.035 | 0.149 |  | 0.457 |
|  | 2*θ* FWHM (amorphous region) | 7.112 ± 0.063 | 7.129 ± 0.292 | 0.062 |  | 0.312 |
|  | *I*_200_*/ I*_120_ | 0.371 ± 0.219 | 0.219 ± 0.318 | 0.267 |  | 0.062 |
|  | X_c_ | 0.481 ± 0.248 | 0.683 ± 0.025 | 0.443 |  | 0.003* |
|  | f_c_ | 0.768 ± 0.406 | 0.774 ± 0.599 | 0.090 |  | 0.083 |
| (b) *Eriophora transmarina* |  |  |  |  | 0.120 |  |
|  | *L* | 10.647 ± 1.951 | 9.535 ± 1.520 | 0.157 |  | 0.061 |
|  | 2*θ* FWHM (200) | 3.033 ± 0.184 | 3.239 ± 0.110 | 0.147 |  | 0.290 |
|  | 2*θ* FWHM (120) | 1.581 ± 0.059 | 1.641 ± 0.055 | 0.094 |  | 0.393 |
|  | 2*θ* FWHM (amorphous region) | 6.423 ± 0.105 | 7.118 ± 0.165 | 0.221 |  | 0.096 |
|  | *I*_200_*/ I*_120_ | 0.231 ± 0.111 | 0.209 ± 0.023 | 0.059 |  | 0.463 |
|  | X_c_ | 0.521 ± 0.204 | 0.707 ± 0.094 | 0.141 |  | 0.097 |
|  | f_c_ | 0.437 ± 0.102 | 0.498 ± 0.155 | 0.076 |  | 0.540 |
| (c) *Latrodectus hasselti* |  |  |  |  | 0.083 |  |
|  | *L* | 8.870 ± 0.620 | 8.690 ± 0.170 | 0.267 |  | 0.506 |
|  | 2*θ* FWHM (200) | 2.203 ± 0.092 | 2.287 ± 0.103 | 0.312 |  | 0.357 |
|  | 2*θ* FWHM (120) | 1.511 ± 0.132 | 1.533 ± 0.060 | 0.236 |  | 0.299 |
|  | 2*θ* FWHM (amorphous region) | 7.718 ± 0.391 | 7.135 ± 0.150 | 0.090 |  | 0.318 |
|  | *I*_200_*/ I*_120_ | 0.209 ± 0.019 | 0.167 ± 0.028 | 0.282 |  | 0.107 |
|  | X_c_ | 0.728 ± 0.041 | 0.726 ± 0.269 | 0.174 |  | 0.234 |
|  | f_c_ | 0.910 ± 0.289 | 0.818 ± 0.517 | 0.128 |  | 0.157 |
| (d) *Nephila plumipes* |  |  |  |  | 0.011* |  |
|  | *L* | 7.235 ± 0.124 | 8.287 ± 0.081 | 0.327 |  | 0.002* |
|  | 2*θ* FWHM (200) | 3.262 ± 0.349 | 2.904 ± 0.127 | 0.190 |  | 0.089 |
|  | 2*θ* FWHM (120) | 1.558 ± 0.018 | 1.578 ± 0.466 | 0.019 |  | 0.602 |
|  | 2*θ* FWHM (amorphous region) | 8.183 ± 0.107 | 6.751 ± 0.112 | 0.475 |  | 0.016* |
|  | *I*_200_*/ I*_120_ | 0.287 ± 0.020 | 0.385 ± 0.043 | 0.186 |  | 0.110 |
|  | X_c_ | 0.554 ± 0.105 | 0.587 ± 0.132 | 0.141 |  | 0.113 |
|  | f_c_ | 0.235 ± 0.751 | 0.272 ± 0.385 | 0.221 |  | 0.077 |
| (e) *Phongnatha graeffei* |  |  |  |  | 0.159 |  |
|  | *L* | 9.733 ± 0.943 | 10.873 ± 1.322 | 0.158 |  | 0.223 |
|  | 2*θ* FWHM (200) | 2.850 ± 0.087 | 2.989 ± 0.159 | 0.269 |  | 0.257 |
|  | 2*θ* FWHM (120) | 1.664 ± 0.076 | 1.682 ± 0.489 | 0.124 |  | 0.289 |
|  | 2*θ* FWHM (amorphous region) | 7.405 ± 0.217 | 7.199 ± 0.128 | 0.103 |  | 0.365 |
|  | *I*_200_*/ I*_120_ | 0.864 ± 0.585 | 0.782 ± 0.290 | 0.204 |  | 0.224 |
|  | X_c_ | 0.589 ± 0.362 | 0.353 ± 0.125 | 0.345 |  | 0.087 |
|  | f_c_ | 0.464 ± 1.014 | 0.505 ± 0.754 | 0.298 |  | 0.239 |
