# Supplementary material for: Multiscale mechanisms of nutritionally induced property variation in spider silks
Source: PLoS One. 2018 Feb 1;13(2):e0192005. doi: 10.1371/journal.pone.0192005 (PMC5794138; doi:10.1371/journal.pone.0192005)
Supplement: S5 Table — Contains statistics for five (one per species) single-factor multivariate analyses of variance. (DOCX) [file pone.0192005.s005.docx]

**S5 Table.** Means (±S.E) mole percentage compositions of the amino acids glutamine, serine, proline, glycine, and alanine, across the protein fed and protein deprived feeding treatments. Adjusted R^2^ statistics for a sum-of-squares (SS) for the whole model against SS for the residuals model are shown as estimates of the proportion of variance explained. Statistics for the five (one per species) single-factor multivariate analyses of variance (Wilk’s λ, with * denoting significance variations across treatments at α < 0.05) are shown, as are P-values are for Fisher’s Least Significant Difference tests (with * denoting significance differences between treatments).

|  |  | Treatment (means ±SE) | | Adjusted R^2^ (SS Whole model vs SS Residuals) | Wilk’s λ (d.f. =5,10) | Fisher’s P-value |
| --- | --- | --- | --- | --- | --- | --- |
| (a) *Argiope keyserlingi* | Amino acid | Protein deprived | Protein fed | Adjusted R^2^ | 0.002* |  |
|  | Glutamine | 9.634 ± 1.058 | 11.633 ± 0.272 | 0.108 |  | 0.103 |
|  | Serine | 5.867 ± 0.043 | 6.768 ± 1.003 | 0.179 |  | 0.126 |
|  | Glycine | 32.807 ± 3.816 | 39.233 ± 0.196 | 0.472 |  | 0.002* |
|  | Alanine | 22.967 ± 3.867 | 30.001 ± 0.158 | 0.387 |  | 0.012* |
|  | Proline | 7.086 ± 1.921 | 10.307 ± 3.100 | 0.317 |  | 0.062 |
| (b) *Eriophora transmarina* |  |  |  |  | 0.494 |  |
|  | Glutamine | 8.033 ± 1.822 | 7.967 ± 0.328 | 0.129 |  | 0.508 |
|  | Serine | 5.533 ± 0.285 | 5.867 ± 5.970 | 0.150 |  | 0.430 |
|  | Glycine | 41.367 ± 0.105 | 41.098 ± 1.433 | 0.051 |  | 0.432 |
|  | Alanine | 24.933 ± 1.998 | 24.377 ± 0.968 | 0.125 |  | 0.462 |
|  | Proline | 6.900 ± 1.903 | 6.055 ± 1.168 | 0.052 |  | 0.683 |
| (c) *Latrodectus hasselti* |  |  |  |  | 0.320 |  |
|  | Glutamine | 13.201 ± 1.058 | 11.633 ± 0.272 | 0.107 |  | 0.274 |
|  | Serine | 5.567 ± 0.470 | 6.667 ± 1.003 | 0.124 |  | 0.442 |
|  | Glycine | 38.180 ± 2.122 | 35.933 ± 1.476 | 0.216 |  | 0.176 |
|  | Alanine | 20.806 ± 1.212 | 21.467 ± 1.549 | 0.151 |  | 0.214 |
|  | Proline | 5.333 ± 1.020 | 5.936 ± 0.088 | 0.006 |  | 0.312 |
| (d) *Nephila plumipes* |  |  |  |  | 0.236 |  |
|  | Glutamine | 8.508 ± 1.967 | 6.057 ± 1.870 | 0.306 |  | 0.052 |
|  | Serine | 3.704 ± 0.321 | 4.033 ± 0.384 | 0.152 |  | 0.389 |
|  | Glycine | 40.843 ± 0.796 | 41.008 ± 1.209 | 0.351 |  | 0.086 |
|  | Alanine | 29.443 ± 2.040 | 33.206 ± 1.677 | 0.459 |  | 0.055 |
|  | Proline | 4.533 ± 1.968 | 1.776 ± 1.468 | 0.517 |  | 0.003* |
| (e) *Phongnatha graeffei* |  |  |  |  | 0.494 |  |
|  | Glutamine | 9.201 ± 0.400 | 5.682 ± 1.153 | 0.245 |  | 0.473 |
|  | Serine | 5.543 ± 0.276 | 5.607 ± 0.104 | 0.179 |  | 0.328 |
|  | Glycine | 41.967 ± 3.448 | 43.176 ± 2.284 | 0.206 |  | 0.223 |
|  | Alanine | 24.067 ± 1.048 | 27.106 ± 1.968 | 0.247 |  | 0.432 |
|  | Proline | 11.133 ± 0.890 | 5.550 ± 1.862 | 0.536 |  | 0.001* |
