# Supplementary material for: Multiscale mechanisms of nutritionally induced property variation in spider silks
Source: PLoS One. 2018 Feb 1;13(2):e0192005. doi: 10.1371/journal.pone.0192005 (PMC5794138; doi:10.1371/journal.pone.0192005)
Supplement: S2 Fig — (DOCX) [file pone.0192005.s007.docx]

**S2 Figure. Examples of WAXS derived MA silk intensity *vs* azimuthal angle plots at the (120) diffraction peaks.**

Where: a = *Argiope keyserlingi*, b = *Eriophora transmarina*, c = *Latrodectus hasselti*, d = *Nephila plumipes*, e = *Phonognatha graeffei*.
