# Supplementary material for: Multiscale mechanisms of nutritionally induced property variation in spider silks
Source: PLoS One. 2018 Feb 1;13(2):e0192005. doi: 10.1371/journal.pone.0192005 (PMC5794138; doi:10.1371/journal.pone.0192005)
Supplement: S3 Fig — Includes both protein deprived and protein fed spiders. (DOCX) [file pone.0192005.s008.docx]

MaSp1a

**S3 Figure.** **Melt curves for MaSp1a and MaSp2a for the five species.**

Includes both protein deprived and protein fed spiders. Where: Ak = *Argiope keyserlingi*, Et = *Eriophora transmarina*, Lh = *Latrodectus hasselti*, Np = *Nephila plumipes*, Pg = *Phonognatha graeffei*.
